# Neuroactive Steroids: Receptor Interactions and Responses

**DOI:** 10.3389/fneur.2017.00442

**Published:** 2017-08-28

**Authors:** Kald Beshir Tuem, Tesfay Mehari Atey

**Affiliations:** ^1^Department of Pharmacology, School of Pharmacy, College of Health Sciences, Mekelle University, Mekelle, Ethiopia; ^2^Clinical Pharmacy Unit, School of Pharmacy, College of Health Sciences, Mekelle University, Mekelle, Ethiopia

**Keywords:** neuroactive, steroids, receptors, interactions, responses

## Abstract

Neuroactive steroids (NASs) are naturally occurring steroids, which are synthesized centrally as *de novo* from cholesterol and are classified as pregnane, androstane, and sulfated neurosteroids (NSs). NASs modulate many processes *via* interacting with gamma-aminobutyric acid (GABA), *N*-methyl-d-aspartate, serotonin, voltage-gated calcium channels, voltage-dependent anion channels, α-adrenoreceptors, X-receptors of the liver, transient receptor potential channels, microtubule-associated protein 2, neurotrophin nerve growth factor, and σ1 receptors. Among these, NSs (especially allopregnanolone) have high potency and extensive GABA-A receptors and hence demonstrate anticonvulsant, anesthetic, central cytoprotectant, and baroreflex inhibitory effects. NSs are also involved in mood and learning *via* serotonin and anti-nociceptive activity *via* T-type voltage-gated Ca^2+^ channels. Moreover, they are modulators of mitochondrial function, synaptic plasticity, or regulators of apoptosis, which have a role in neuroprotective *via* voltage-dependent anion channels receptors. For proper functioning, NASs need to be in their normal level, whereas excess and deficiency may lead to abnormalities. When they are below the normal, NSs could have a part in development of depression, neuro-inflammation, multiple sclerosis, experimental autoimmune encephalitis, epilepsy, and schizophrenia. On the other hand, stress and attention deficit disorder could occur during excessive level. Overall, NASs are very important molecules with major neuropsychiatric activity.

## Overview of Neuroactive Steroids (NASs)

Neuroactive steroids are types of steroids—which are occurring naturally—in which they have an impact on behavioral actions, change excitability of neurons, and results in non-genomic effects and through the interaction with specific neurotransmitter receptors (1, 2). Adrenal glands, ovary, and brain are their sites of production either from cholesterol or *via* metabolism of deoxycorticosterone, testosterone, and progesterone—which are their blood-borne precursors (3). Moreover, they are also produced in fetoplacental unit (4). The term “neurosteroids” (NSs) are coined since cholesterol is the precursor for *de novo* synthesis of NSs centrally (5).

Grossly, NASs can be categorized into three classifications, namely, pregnane NSs, androstane NSs, and sulfated NSs. The pregnane NSs consisted of progesterone derivatives such as allopregnanolone (ALLO) (3α, 5α-tetrahydroprogesterone), epiallopregnanolone (3β, 5α-tetrahydroprogesterone), pregnanolone (3α, 5β-tetrahydroprogesterone), pregnenolone (PREG), dehydroepiandrosterone (DHEA), and allotetrahydrodeoxycorticosterone (THDOC). The second classification consisted of androstane NSs including both androstanediol and etiocholanone and last sulfated NSs comprised dehydroepiandrosterone sulfate (DHEAS) and pregnenolone sulfate (PREGS) (6, 7). Furthermore, vitamin D is categorized as NSs as it affects the brain of younger children and adult population (8).

Dehydroepiandrosterone acts as an antagonist of cortisol and is the most plentiful circulating steroid among the NASs in human being (9). The sulfated form of this NASs—DHEA-S—has a relatively long half-life and in animal models, DHEA-S enhances cognitive and behavioral performance (10). Androstenol is a special type of NSs, acts as a pheromone, and has a higher structural resemblance to gamma-aminobutyric acid-A (GABA-A) receptor modulating NSs and as a result of this, act as a signaling molecule between entities of the alike species through interaction with GABA-A receptors (11).

Neurosteroids have revealed contribution in numerous nueropathophysiological processes, including aggression, mood, energy, general activity, learning, and memory processes (12), excitatory or inhibitory effects of different neurotransmitters, upsurge serotonin levels, and the inhibitory action against certain cortisol effects in the brain (12, 13). To realize their role, NSs involve allosteric modulation on GABA (14), *N*-methyl-d-aspartate (NMDA) glutamate (15), serotonin (5-HT3) (16), and alpha-1 receptors (17).

## Biosynthesis of NASs

Reduction of the A-ring from testosterone, deoxycorticosterone, and progesterone—which are steroid hormones—results in the formation of NSs (4). These chemicals can be released into the blood and act systemically or synthesized *de novo* locally, from cholesterol (5, 18), in certain brain parts such as in the pineal gland (the major site for neurosteroidogenic organ), cortex, glutamatergic neurons, hippocampus, and cortex (19).

Allopregnanolone and 7α-OH PREG were exceedingly produced and show vital roles in the Purkinje cell facilitation of survival in the juvenile quail (20) by suggested action *via* GABA-A receptor (21). Moreover, 3α-hydroxysteroid oxidoreductase (3α-HSOR) and 5α-reductase enzymes reduce the precursor steroid—found peripherally in skin and liver—to produce androstanediol, ALLO, and THDOC (22). Other substances are also involved in the induction of the biosynthesis of NASs: retinoic acids and vitamin D3 (VD3) induce neurosteroid production in human glial cells in culture (23), VD3 *via* induction of CYP11A1 and HSD3B1 (steroidogenic genes), which is mediated by vitamin D receptor (24).

**Figure 1:**
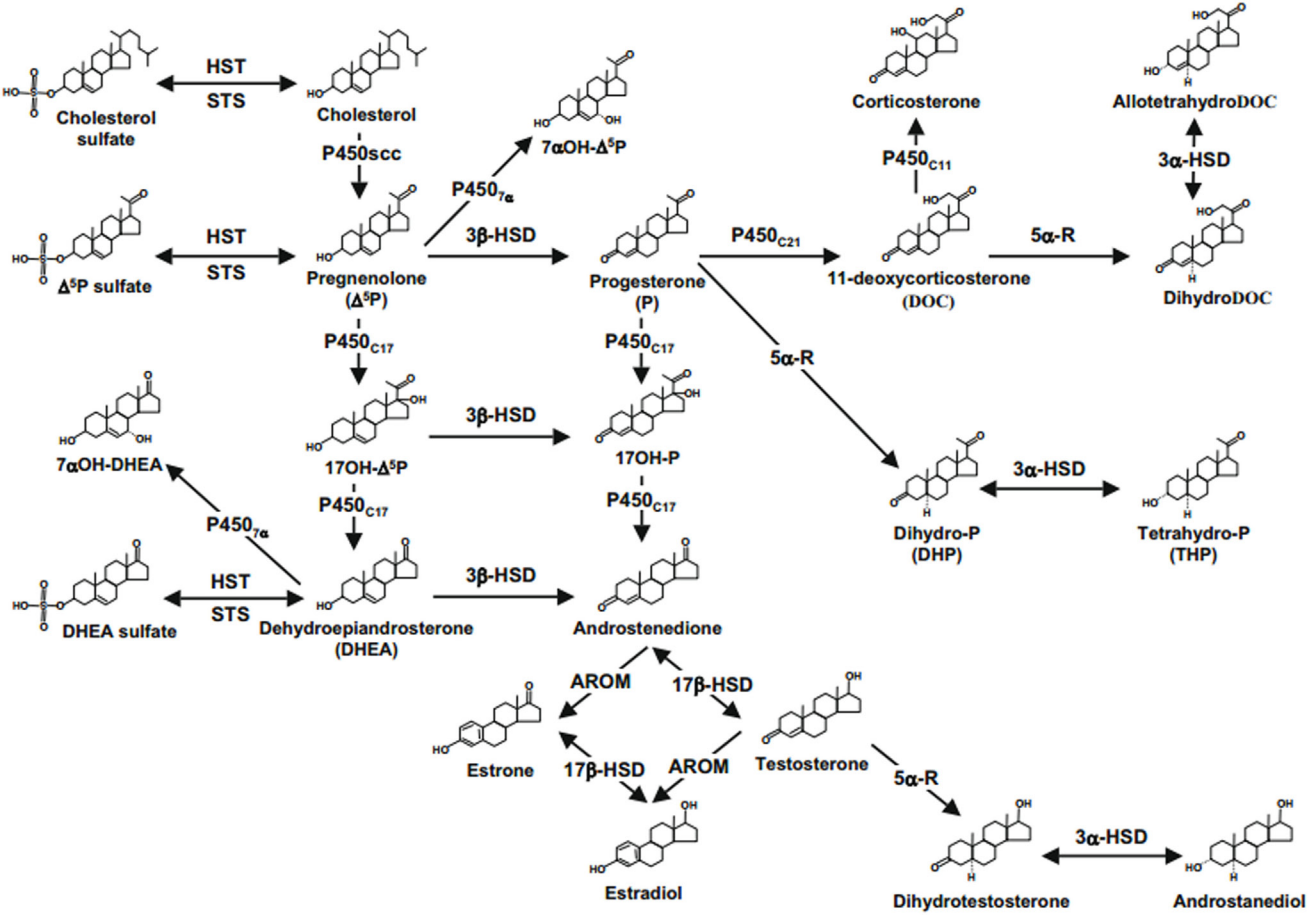
Neurosteroids biosynthesis in the central nervous system. Abbreviations: 17b-HSD, 17b-hydroxysteroid dehydrogenase; 3a-HSD, 3a-hydroxysteroid dehydrogenase; 3b-HSD, 3b-hydroxysteroid dehydrogenase; 5a-R, 5a-reductase; AROM, aromatase; HST, sulfotransferase; P4507a, cytochrome 7a-hydroxylase; P450C11b,11b-hydroxylase; P450C17, cytochrome P450 17a-hydroxylase/C17,20-lyase; P450C21, 21-hydroxylase; P450scc, cytochrome P450 side-chain cleavage; STS, sulfatase (2, 22, 61).

Some factors affect the biosynthesis of NASs. The mRNA expression of 5α-reductase type I (5α-RI) is markedly downregulated (~50%) by social isolation in neurons of the cortex, hippocampus, and basolateral nucleus of the amygdala. For instance, 65–75% and smaller (~35%) decrease of 5α-RI mRNA levels were observed in dentate gyrus granule cells and CA3 glutamatergic pyramidal neurons, and frontal cortex pyramidal neurons (layer V/VI glutamatergic), respectively. Therefore, the anxiety and aggressive behavior seen in mice, which is socially isolated, is due to decreased ALLO biosynthesis in glutamatergic neurons of basolateral nucleus of the amygdala and frontal cortex (25) (Figure 1).

## Mechanisms of Action of NASs

The mechanism of action of NASs can be classified as either (a) classical intracellular binding—in which this effect is described as a relatively slow genomic effects of NASs, (b) effect on membrane receptors and ion channels—in which this effect is also described as a rapid non-genomic effects of NASs, or (c) due to their metabolic interconversion to traditional steroids in the brain whereas some steroids led to rapid membrane effects through interaction with certain neurotransmitter receptors (6, 26). In summary, NSs predominantly interact with ion channels and neuronal membrane receptors—but not primarily through interaction with intracellular receptors—and thereby modulate brain excitability (27), preferably through direct modulation of ion channels that are ligand-gated, remarkably GABA-A receptors (28).

## Regulation of NASs

In the supraoptic nucleus, oxytocin regulates neurosteroid modulation of GABA-A receptors after parturition, since the activity of protein kinase C (PKC) and sensitivity of GABA-A receptor to ALLO in the supraoptic nucleus are mainly determined by the magnitude of activation of oxytocin receptor. Besides this, the GABA-A receptors are ALLO-resistant in breastfeeding mothers due to the presence of high level of oxytocin (29). The presence of relatively high concentrations of endogenous phosphatase during late pregnancy enhances the sensitivity of ALLO to GABA-A receptors. On the other hand, sensitivity of ALLO is restored by these phosphatases stimulation—which are endogenous—or PKC inhibition when NSs are becoming insensitive to GABA-A receptors (29). Instead, effect of the endogenous phosphatases and PKC on dissimilar phosphorylation sites or varying proteins of GABA, which are found on postsynaptic sites, may be prominent (30).

## Metabolism of NASs

Neuroactive steroids undergo multiple stages for their metabolism. The brain microsomes process the two main NSs—PREG and DHEA—and convert them into their corresponding 7α-hydroxylated derivatives. Consequently, the concentration of active metabolites may be regulated by the production of these 7α-hydroxylated derivatives of PREG (progesterone) (31) and DHEA (androstenedione, a precursor of testosterone) (32). Furthermore, detection of a little amount of 7 beta-OH metabolites of DHEA and PREG has been possible, but their characterization was not definite (33).

## Effect of NASs

### Effect of NSs on Receptors of Gamma-Aminobutyric Acid

#### Effect on Gamma-Aminobutyric Acid-A Receptors

Gamma-aminobutyric acid-A receptors (GABA-A receptors) are major targets for central nervous system (CNS) actions of NSs (34). The GABA-A receptor function can be regulated by the NSs negatively or positively, based on the chemical structure of the steroid molecule (27, 35). Secondary to the activation of the inotropic GABA-A receptors by NASs, chloride ion influx and causes neuronal membrane hyperpolarization (36).

The GABA-A receptors have novel subunit dependence of NSs action (34). The neurosteroid THDOC favorably augment the receptor with σ subunit among the seven different classes of subunits (α1–6, β1–3, γ1–3, σ1–3, δ, ε, θ). High (micromolar) and low (nanomolar) levels of NSs cause direct activation of GABA-A receptors and allosteric augmentation of GABA-regulated currents, respectively (37). However, changing in the modulation of NSs and inhibition of intact GABA are observed in neurons found in thalamic relay of mice without delta subunit (34). Inhibition of PKC activity abolishes the effect of THDOC on GABA-A receptors through increased α-4 subunit phosphorylation and its accumulation on cell surface by accelerating the α-4 subunit insertion into cell membrane without altering their endocytosis (38). The increased effect of THDOC on the phosphorylation of α-4 subunit and its expression on the cell surface is counteracted by S443 mutation, which is the major site of phosphorylation of PKC in the α-4 subunit (39).

Neurosteroids increased expression of α_4_βγ_2_ GAB-A receptors at Cornu Ammo (CA1) pyramidal cell synapses. The diminution in decay time for GABAergic miniature inhibitory postsynaptic currents following short-term NSs exposure is mediated by this increased expression—containing α4-GAB-A receptors localized to synaptic sites (40).

During stress, the balance between inhibition and excitation may be maintained by NSs, in which GABA-A receptor regulation is influenced by the levels of and duration of exposure to NSs (41). The plasma level of THDOC is elevated by about three times in the presence of stress (42). From the NASs, ALLO, THDOC, and androstanediol are powerful modulators of the GABA-A receptor (through positive allosteric modulation) and bring behavioral effects at low concentrations (1, 43, 44). Allopregnanolone was fully effective in suppressing GnRH release, mediated by interaction with the GABA-A receptor. Moreover, ALLO suppressive action on GnRH release *in vitro* is completely offset by GABA-A antagonistic NASs, PREG-S (31).

Enhancement of NSs due to reduced sensitivity of GABAergic synaptic transmission in dentate granule cells lead to the blockage of seizure propagation into the hippocampus (45). In women, seizure exacerbation during the perimenstrual period is contributed by the loss of NSs sensitivity of synaptic inhibition. In addition, withdrawal and elevated concentrations of NSs—observed in the mid-cycle—leads to exacerbation of seizure and anticonvulsant action, respectively (46). In the spinal cord, obliteration of induction of activity-dependent reflex plasticity is mediated by modulation of GABA-A receptors dependent inhibition as a result of the activity of the progesterone and its metabolites (ALLO and THDOC) (47).

Neurosteroids are potent cytoprotectants when they interact with neuronal GABA-A receptor. The potency and efficacy of NSs is affected by their structure. Sulfated NSs (PREGS and DHEAS) fully efficacious (about 70%) than the non-sulfated (AP and 17α-OH-AP). This is further substantiated by the structural activity analysis that indicated the association of an increase in potency but decrease in efficacy of the cytoprotectants with the lack of the double bond between C-5 and C-6 in AP and 17α-OH-AP and/or the hydroxyl group in the α-position (48).

Dehydroepiandrosterone and its sulfated metabolite, DHEA-S (more potent and more efficacious than the parent compound), which interacted with the picrotoxin/TBPS (t-butylbicyclophosphorothionate) binding site in a competitive manner (49), decreased GABA-A receptor-mediated responses on serotonin (5-HT) neuronal firing regulation, and *vice versa*. Androsterone and its parent compound (DHEA) can affect anxiety, cognition, and mood through enhancement of the GABA-A mediated response (50). Inhibition of NMDA receptor and potentiation of GABA-A receptor function, which will add on the clinical profile of anesthetic NASs—is mediated by another neurosteroid compound: (3α, 5β)-20-oxo-pregnane-3-carboxylic acid (3α5βPC). Notwithstanding, a NAS with a better clinical activity can be produced by augmenting blockage of NMDA receptor and reducing GABA-A receptor blockage since a direct correlation of the optimal property of anesthetic, anticonvulsant, and neuroprotective with high micromolar concentrations of 3α5βPC—needed for blocking effect on GABA-A receptors—might not be observed (51).

Stereospecific non-genomic activity on GABA-A receptor results in sharp increases in blood concentrations of 3α-OH-DHP—the neuroactive metabolite of progesterone—and ultimately leads to enhancing the baroreflex inhibition of brainstem rostral ventrolateral medulla neurons (52).

#### Effect on Gamma-Aminobutyric Acid-C Receptors

The interaction of NASs with the ρ1-GABA-C receptor—which is very specific chiral sites—and 3α configuration of GABA-C receptor is required for all steroid actions. NSs have multiple sites for interaction with the ρ1 receptor of GABA-C than GABA-A. However, a similarity in qualitative measurements and GABA-A receptor potentiation was observed in sites mediating 5β-reduced steroids inhibition and potentiation of the ρ1 receptor by 5α receptors (53).

Allopregnanolone, alphaxalone, and 5α-THDOC prolong the decay time and potentiate the GABA-induced currents. On the contrary, the ρ1-GABA-evoked current is inhibited by the co-administration of GABA with 5β-THDOC, pregnenolone, or 5β-DHP. The degree of inhibition and potentiation of ρ1-GABA provoked currents by NASs is reliant on the concentration of GABA. Since the application of GABA alone, following treatment with NAS, did not revert back to the control level for an extended period of time, a prolonged and persistent effect was observed on the effects of the NASs on ρ1 receptor channels. The 5α derivatives were potentiators (only at exceedingly low concentrations of GABA), whereas the 5β compounds were inhibitors of the GABA-evoked currents (54).

### Effect of NSs on *N*-Methyl-d-Aspartate Receptors

Neurosteroids (PS and PHS) control the NMDA receptors dependent (4) and independent (L-type calcium channel-dependent) long-term potentiation (LTP) positively at a lower dose (1–5 mM) and negatively at a higher concentration (15 mM) (sigma-receptor function blockade) (55). Positive modulation is through increased Ca^2+^ influx into presynaptic NMDA receptors (containing NR2D subunits) that raise the probability of glutamate discharge in hippocampal slices as studied in rats less than 6 days age (56, 57). The positive regulation was observed to be negatively affected by antepartum ethyl alcohol exposure due to a change in NMDA receptor phosphorylation (58).

**Figure 2:**
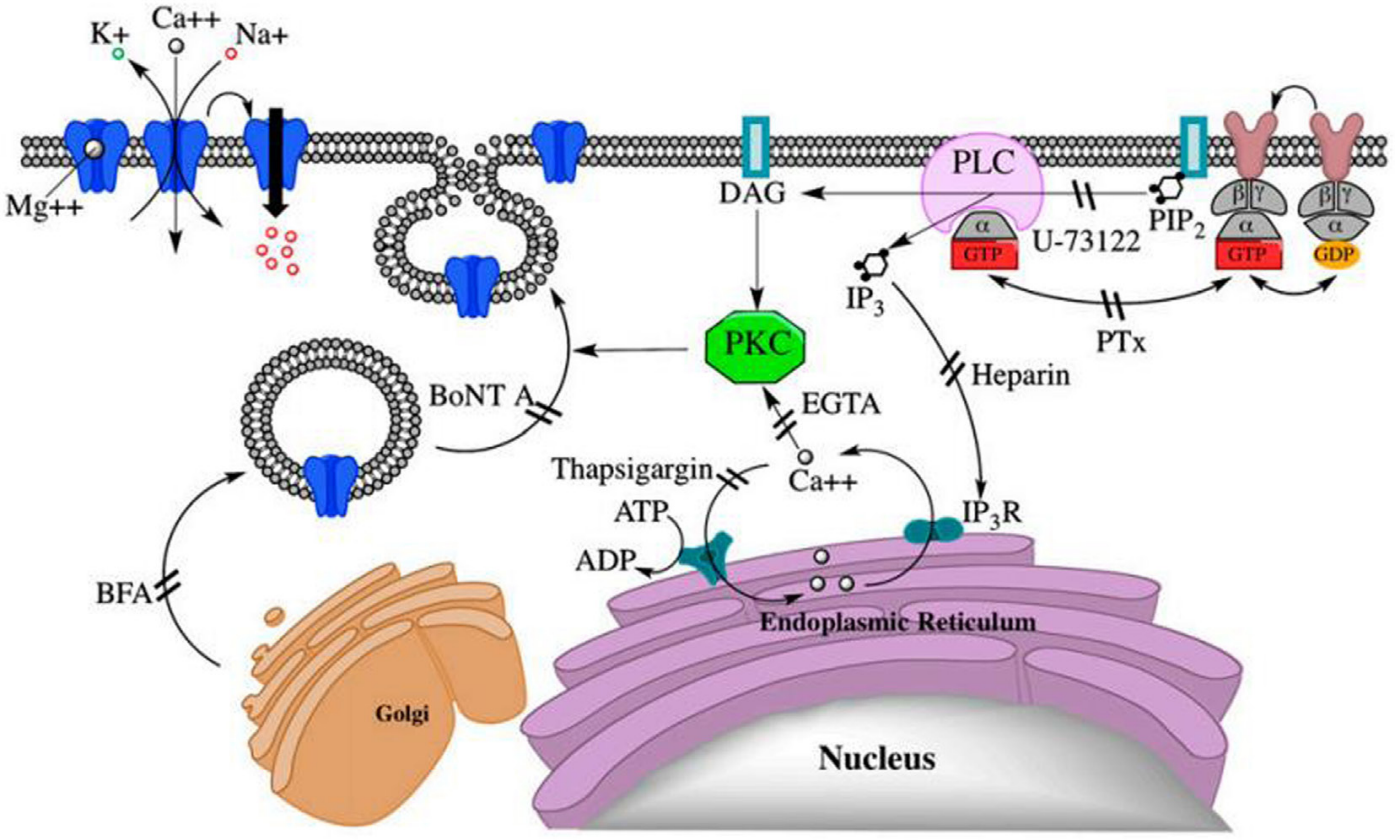
PregS-stimulated trafficking of functional *N*-methyl-d-aspartate (NMDA) receptors to the cell surface *via* a non-canonical G protein-, PLC-, Ca2^+^-, and protein kinase C (PKC)-dependent mechanism. DAG, diacylglycerol; IP3R, IP3 receptor; NMDA, (*N*)methyl-d-aspartate; PIP2, phosphatidylinositol 4,5-bisphosphate (59).

Pregnenolone sulfate stimulates a continued increase in the NMDA effect from 200 to 400%, by integration of extra subunit specific (σ1) receptors into the surface membrane (57, 59). This integration requires G-protein-coupled activation of PKC and PLC and increased Ca^2+^ ion (Figure 2) (59). The movement of NMDA receptors between the membrane and intracellular pools is required for maintenance and plasticity of synaptic connections; however, deregulation of the receptor movement has been associated with neuropsychiatric disorders (60). Hence, NMDA receptor surface expression plays a great role in disorders of NMDA receptor trafficking or provides a basis for the development of therapeutic interventions (59).

### Effect of NSs on Voltage-Gated Calcium Channel Receptors

#### Effect on L-Type Voltage-Gated Calcium Channels

Allopregnanolone (APα) (3α-hydroxy-5α-pregnan-20-one) promotes proliferation of hippocampal neural progenitor cells in rat and cerebral cortical neural stem cells (NSCs) in human at a nanomolar levels, and inhibits the proliferation of polysialylated form of the neural cell adhesion molecule (PSA-NCAM) at micromolar levels (62). This mechanism requires activation of L-type voltage-gated Ca^2+^ channels (VGLCCs) (63). In mature neurons, APα increases chloride influx *via* allosteric modulation of the GABA-A receptor, thereby hyperpolarizing the neuronal membrane potential and decreasing neuron excitability (36). In marked contrast, it leads to an efflux of chloride in immature neurons, which causes depolarization of the membrane and opening of VGLCCs, then rises in intracellular Ca^2+^ (63, 64). Finally, this can trigger calcium-dependent mechanisms of mitosis in early precursor cells and human NSCs to promote neurogenesis (65).

#### Effect on T-Type Voltage-Gated Calcium Channels

The 5β-reduced NASs are powerful inhibitors of the T-type Ca^2+^ channels in rat peripheral sensory neurons *in vitro* and very effective peripheral anti-pain agents *in vivo*, strongly proposing that T-type Ca^2+^ channels are involved in peripheral somatic nociception (66). Thus, the 5β-reduced steroids are hopeful new agents for studying the role of T-type Ca^2+^ channels in peripheral nociception and are potentially useful targets for the development of new pain therapies (67).

### Effect of NSs on Serotonin Receptor

Neurosteroids (DHEA) interact with ligand-gated serotonin (5-HT) receptors to enhance their firing action through σ1 receptors (68). This stimulates neurogenesis in the hippocampal dentate gyrus and shields it from glucocorticoids’ detrimental attack (69).

Since DHEAs interact with σ1 receptors to bring their effect, σ1 receptor antagonists could eliminate the effect of DHEAs, whereas agonists of the receptor could simulate the blocking effect of DHEAs on 5-HT-evoked glutamate release *via* activation of Gi protein pyramidal cells of rat prelimbic cortex. At a lower concentration (1 µM), DHEAs could significantly hinder the 5-HT-evoked glutamate release in brain region by preventing its binding with 5-HT3. In reverse, DHEAs effect can be lessened with the rise in concentration due to promoting spontaneous glutamate release (70).

There is a relationship between central serotonergic activity and circulating ALLO concentrations. This is evidenced by an increase in ALLO concentrations in luteal phase after the administration of l-tryptophan in both controls and women with premenstrual syndrome (PMS) (greater increase in ALLO concentrations with PMS) (71).

### Effect of NSs on α_2_-Adrenergic Receptors

Neurosteroids (PREGS) blocks LTP of excitatory synapses in rat medial prefrontal cortex (mPFC) *via* interaction with α_2_-adrenoreceptors secondary to enhancement of Gi proteins. After treatment of mPFC slices with the α_2_-adrenoreceptor inhibitor “yohimbine,” the blocking effect of PREGS on the induction of LTP was completely inhibited (72).

### Effect of NSs on Transient Receptor Potential Channels

Mammalian transient receptor potential melastatin (TRPM) proteins—such as TRPM1 and TRPM3—get together into ion transporting canals and respond to temperature, osmolarity, various chemical signals, change in membrane voltage, oxidative stress, and intracellular calcium (73). PREGS, pregnenolone, and epipregnanolone sulfate were found to potentiate TRPM3 activity. As compared to pregnenolone, PREGS shows comparable potency but greater than 10-fold higher intrinsic activity (74, 75), and it stimulates TRPM3 activity *via* heat-dependent modulation (76).

### Effect of NSs on Reward Pathway *via* the σ1 Receptor

The σ1 receptor is an intracellular neuronal protein found in endoplasmic reticular, plasma, nuclear, and mitochondrial membranes. The ligands of σ1 receptor potently modulate intracellular Ca^2+^ mobilizations and extracellular Ca^2+^ influx (77, 78). Pregnenolone, DHEA, and progesterone interfere with cocaine-induced reward path way in mice. DHEA and its precursor PREG facilitate cocaine-induced conditional place preference acting as σ1 receptor agonists (79).

### Effect of NSs on Liver X Receptors (LXRs)

Liver X receptors have two isoforms, LXRα (NR1H3) and LXRβ (NR1H2), classified under nuclear receptor superfamily. LXRα is highly found in liver and minimally in the intestine, macrophages, adipose tissue, lung, kidney, and adrenal gland; whereas LXRβ is broadly expressed (80). Oxysterols (oxidized cholesterol) bind with LXRs and induce expression of genes which eradicate harmful cholesterol level by efflux through ATP-binding cassette family of transporters (81). Activation of LXRs promote cholesterol disposal, steroidogenesis in the adrenal gland, regulation of StAR expression, restores normal StAR mRNA levels and completely restores the mRNA levels of P450scc to non-diabetic levels, and raises the local levels of NASs like PROG and DHP. These functions play a role in protecting diabetes patients from peripheral neuropathy (82).

### Effect of NSs on Voltage-Dependent Anion Channels (VDAC)

Interactions of NASs with VDAC isoforms (prominent brain protein) are important to regulate mitochondrial function, synaptic plasticity, or apoptosis. A role in avoiding apoptosis explains the neuroprotective actions of the NASs (83).

### Effect of NSs on Microtubule-Associated Protein 2 (MAP2)

Pregnenolone and its synthetic analog—MePREG interacts with MAP2 at an unknown binding site. Then, it stimulates microtubule polymerization (rat brain and from PC12 cells) and the extension of neuritis in pheochromocytoma cells that are exposed to nerve growth factor (NGF) (84).

### Effect of NSs on Neurotrophin NGF Receptors

Acting as a neurotrophic factor, DHEA affects neuronal survival and neurogenesis during development and in aging in the brain *via* interacting with pro-survival TrkA and pro-death p75 (NTR) membrane receptors of neurotrophin NGF. This stimulation inhibits the apoptotic loss of NGF receptor positive sensory and sympathetic neurons. Furthermore, it provides a mechanistic explanation for the multiple actions of DHEA in other peripheral biological systems expressing NGF receptors, such as the immune, reproductive, and cardiovascular systems (85) (Table 1).

**Table 1:** Effect of neurosteroids on various receptors.

Receptor	Neurosteroid	Effects	Reference
GABA-A	THDOC, androstanediol, AP, PREGS, 3α5βPC	Neuronal membrane hyperpolarization, cytoprotectants, anesthetic, anticonvulsant, neuroprotective (+), suppressing GnRH release, blockage of seizure propagation into the hippocampus (−)	(27, 34–36)
GABA-C	AP, 5α-THDOC, 5β-DHP	Potentiation of GABA-induced currents (+) at high concentration by 5α and (−) by 5β	(53, 54)
NMDA	PS, PHS	Raise glutamate discharge in hippocampal slices (+) at lower dose and (−) at higher dose	(55–57)
L-type VGLCCs	APα	Promotes proliferation of hippocampal neural progenitor cells at nanomolar level, promote neurogenesis (+)	(62, 65)
T-type VGLCCs	5β-reduced NASs	Anti-pain agents *in vivo* (−)	(66)
Serotonin	AP, DHEA	Neurogenesis in hippocampal dentate gyrus (+)	(69)
α2-adrenergic	PS	Blocks long-term potentiation (−)	(72)
TRPM	PREGS, PS, epipregnanolone sulfate	Respond to temperature, osmolarity, various chemical signals, change in membrane voltage, oxidative stress, and intracellular calcium (+)	(73–76)
σ1 (Reward)	PEG, DHEA, PR	facilitated an acquisition of cocaine-induced conditioned place preference (+)	(79)
Liver X	DHP	Protecting diabetes patients from peripheral neuropathy (+)	(82)
	PROG		
VDAC	NAS	Regulate mitochondrial function, synaptic plasticity, regulators of apoptosis (+)	(83)
MAP2	PREG, MePREG	stimulate microtubule polymerization (+)	(84)
NGF	DHEA	neuronal survival and neurogenesis, immune, reproductive, cardiovascular systems (+)	(85)

*(+) = potentiation of the receptor, (−) = inhibition of the receptor*.

*3α5βPC, (3α, 5β)-20-oxo-pregnane-3-carboxylic acid; ALLO/AP, allopregnanolone; DHEA, dehydroepiandrosterone; DHEAS, dehydroepiandrosterone sulfate; DHP, dihydroprogesterone; GABA-A, GABA-C, gamma-aminobutyric acid type C; MAP2, microtubule-associated protein 2; NASs, neuroactive steroids; NMDA, *N*-methyl-d-aspartate; PHS, pregnenolone hydro-sulfate; PR, progesterone receptor; PREG, pregnenolone; PREGS/PregS/PS, pregnenolone sulfate; PROG, progesterone; THDOC, tetrahydrodeoxycorticosterone; TRPM, mammalian transient receptor potential melastatin; VDAC, voltage-dependent anion channels; VGLCCs, L-type Voltage-gated Ca^2+^ channels; VGTCCs, T-type voltage-gated Ca^+2^ channels*.

## Involvement of NASs in Neurological Diseases

Epilepsy-related with menstruation (catamenial epilepsy) currently has no specific approved treatments. This kind of epilepsy is as a result of enhanced excitability due to withdrawal of NSs, which in turn leads to upregulation of α4 subunit and linked with upregulation of Egr3 and reduced synaptic inhibition. In addition, neurosteroid withdrawn mice were amazingly less sensitive to the antiseizure effects of diazepam, and progesterone receptor (PR) knockout animals were also less sensitive to the protective actions of diazepam during neurosteroid withdrawal (86). Neurosteroid (ALLO) has broad-spectrum anticonvulsant activity and recently it is approved for investigational use to treat an individual with prolonged super refractory status epilepticus (87). Other NS like APα also has anxiolytic and sedative-hypnotic properties with no indicated toxicological adverse events in healthy human volunteers and in children with refractory infantile spasms (88–90). Further investigations are presently ongoing to identify the neurogenic potential of APα in rodent models of aging and Alzheimer’s disease (AD) (63).

The concentrations of testosterone, cortisol, PROG, DHEA, DHEAS, and estrogen levels have been found to be changed in some patients with schizophrenia. The level of DHEA contrariwise associated with negative symptom severity in drug-free men with first-episode psychosis (91). In major depressive episodes, NS, mainly ALLO and pregnenolone, are found to be diminished in both the cerebrospinal fluid (CSF) and the plasma of untreated patients (25). However, following effective psychopharmacological treatment concentrations of ALLO in depressed patients increase to normal levels. Several findings support the hypothesis of an antidepressant effect of ALLO (92).

Stress-induced groups of rats showed declined amount of DHEA-S, which indicates that DHEA may play an important role in the development of adaptive responses to a stressful event (93). Higher blood level of DHEA and DHEAS were related with less experience of symptoms in attention deficit hyperactivity disorder (ADHD), in particular, hyperactivity symptomatology. However, the effect in ADHD patients remains elusive (94). Alterations in gene expression of the enzymes which synthesize NSs may be involved in the pathology of AD. In early AD, there is an attempt to increase the biosynthesis of NSs and NASs through increased mitochondrial import of cholesterol (95).

The amount of AP and DHEA showed significant reduction in specimens of multiple sclerosis white matter compared to controls. Allopregnanolone has a role in controlling neuro-inflammation and protection of demyelination and axonal loss in models of multiple sclerosis by interacting with GABA-A receptor. Experimental autoimmune encephalitis (EAE) is characterized by disordered neurosteroidogenic machinery, which causes reduced expression of the enzymes (3-alpha-hydroxysteroid dehydrogenase) involved in AP biosynthesis, related with reduced AP levels in the CNS. Since AP has a role in controlling neuro-inflammation and protection of demyelination and axonal loss, it also used for the treatment of EAE associated myelin and axonal injury (96).

Levels of allopregnanolone and 5-DHP were found reduced in the CSF of Parkinson’s disease (PD) patients signifying a part for these progesterone metabolites in the disease. Moreover, the enzymes which synthesize allopregnanolone, mRNA expression of 5-reductase type 1 (SRD5A1) was significantly reduced in peripheral blood mononuclear cells of PD patients, proposing a generalized defect in the enzymatic machinery that regulates the metabolism of progesterone (97).

## Author Contributions

KT and TA conducted the review and edited the manuscript.

## Conflict of Interest Statement

The authors declare that the research was conducted in the absence of any commercial or financial relationships that could be construed as a potential conflict of interest.

## Abbreviations

3α5βPC, (3α, 5β)-20-oxo-pregnane-3-carboxylic acid; 3αHSD, 3alpha-hydroxysteroid dehydrogenases; 3α-HSOR, 3alpha-hydroxysteroid oxidoreductase; 5αR, 5alpha-reductase;5αRI, 5alpha-reductase type I; ALLO/AP, allopregnanolone; CA1, Cornu Ammo; CNS, central nervous system; Cyps, cytochrome P450; DHEA, dehydroepiandrosterone; DHEAS, dehydroepiandrosterone sulfate; DHP, dihydroprogesterone; EAE, experimental autoimmune encephalitis; GABA, gamma-aminobutyric acid; GABA-A, gamma-aminobutyric acid type A; GABA-AR, gamma-aminobutyric acid type A receptor; GABA-C, gamma-aminobutyric acid type C; HSD, hydroxysteroid dehydrogenases; LTP, long-term potentiation; MAP2, microtubule-associated protein 2; mIPSCs, miniature inhibitory postsynaptic currents; NASs, neuroactive steroids; NMDA, *N*-methyl-d-aspartate; NPCs, neural progenitor cells; NSs, neurosteroids; NSCs, neural stem cells; PHS, pregnenolone hydro-sulfate; PKC, protein kinase C; PMS, premenstrual syndrome; PR, progestrone receptor; PREG, pregnenolone; PREGS/PregS/PS, pregnenolone sulfate; PROG, progesterone; StAR, steroidogenic acute regulatory protein; TBPS, t-butylbicyclophosphorothionate; THDOC, tetrahydrodeoxycorticosterone; THP, tetrahydroprogesterone; TRPM, mammalian transient receptor potential melastatin; VD, vitamin D; VDAC, voltage-dependent anion channels; VDR, vitamin D receptor; VGLCCs, L-type voltage-gated Ca^2+^ channels; VGTCCs, T-type voltage-gated Ca^2+^ channels.
